# Screening and Identification of Basement Membrane–Related Gene Signatures for Diagnosis in Keratoconus Through WGCNA and Machine Learning

**DOI:** 10.1155/joph/7107888

**Published:** 2025-06-01

**Authors:** Peiyun Xie, Bowei Yuan, Zhanhao Gu, Rong Li, Ding Chen

**Affiliations:** ^1^Eye Hospital, Wenzhou Medical University, Wenzhou 325027, China; ^2^Affiliated Qingyuan Hospital, Qingyuan People's Hospital, Guangzhou Medical University, Qingyuan 511518, Guangdong, China

**Keywords:** basement membrane, bioinformatic analysis, biomarker, keratoconus, machine learning, WGCNA

## Abstract

**Purpose:** Keratoconus (KC) can lead to severe vision loss, impacting daily life. The etiology of KC is not yet clear, and early diagnosis and treatment are crucial for prognosis. This study aimed to explore basement membrane (BM)–related gene signatures for the diagnosis and therapy of KC and provide novel insights into its pathogenesis.

**Methods:** Based on the public datasets GSE112155 and GSE151631 in the GEO database, we obtained the differentially expressed genes (DEGs) of KC and downloaded BM-related genes based on the GeneCards database. Through a combination of bioinformatics methods, primarily weighted gene coexpression network analysis (WGCNA) and machine learning such as random forest (RF) and support vector machine (SVM), BM-related genes were identified as biomarkers for KC diagnosis. Subsequently, we further validated these findings using unsupervised clustering analysis, nomogram, and ROC curve analysis.

**Results:** Through the analysis of two KC-related datasets, 227 DEGs were screened out and intersected with BM-related genes to obtain 195 intersecting genes. By applying WGCNA and two machine learning algorithms, we identified four key genes, namely, CRY2, RNF19B, PPP1R18, and PFKFB3. These genes were significantly expressed in the normal control group. According to the ROC analysis, all four genes demonstrated excellent diagnostic performance in internal validation, with AUC values all exceeding 0.8. In external validation, CRY2, RNF19B, and PPP1R18 showed good predictive performance, each with AUC values greater than 0.6. Unsupervised clustering and nomogram also supported the good diagnostic capabilities of these genes. In addition, unsupervised clustering analysis also indicated that these four genes were mainly distributed in subtype A of KC. Immune infiltration analysis and functional enrichment analysis further suggested that immune inflammation, metabolism, and apoptosis were also involved in KC.

**Conclusion:** Using bioinformatics analysis, we found three novel hub genes, CRY2, RNF19B, and PPP1R18, which are beneficial for the diagnosis and therapy of KC.

## 1. Introduction

Keratoconus (KC) is a bilateral and asymmetric disease, characterized by progressive thinning and ectasia of the central or paracentral cornea, which results in irregular astigmatism and visual loss [1]. It is reported that the prevalence of KC in the general population is about 1.3/1000, especially affecting adolescents [2], while KC in children is more aggressive [3], with a greater impact on vision and daily life. As a result, early identification, which plays a crucial role in stabilizing disease progression and improving patient outcomes, is particularly important for this group. However, there are often no obvious ocular signs in the early stages. And there is no single definitive marker that can clearly distinguish it from normal corneal tissue [4]. So, screening and diagnosing subclinical KC continue to be limited and challenging. The etiology of KC is still uncertain. Recently, some domestic and international studies have reported potential loci and differentially expressed genes (DEGs) relevant to the pathogenesis of KC, such as VSX1, COL4A3, TIMP3, TGFBI, and ZNF469 [5–7]. However, it should be noted that there is no significant overlap among these DEGs, and some potential targets have yet to be consistently validated in subsequent investigations. The treatment options for KC include spectacle frames, contact lenses, corneal cross-linking (CXL), corneal transplantation, stem cell therapy, and so on, but they all have certain limitations [8–10].

The basement membrane (BM) is a thin, amorphous connective tissue structure, classified as a specialized form of extracellular matrix found on the basal aspect of epithelial and endothelial tissues [11–13]. It primarily consists of Type IV collagen, laminin, fibronectin, heparin sulfate proteoglycans, and nidogens [14]. BM serves multiple functions. Firstly, it provides adhesion for epithelial, endothelial, or parenchymal cells, anchors them to underlying connective tissues, and provides essential structural support. Secondly, it regulates cellular functions by modulating the local concentrations of growth factors and cytokines, thereby influencing processes such as cell proliferation, migration, differentiation, fibrosis, and other physiological activities. Moreover, it exhibits barrier properties and participates in signal transduction pathways [15–18]. In general, although previous studies have found that BM is involved in a variety of physiological and pathological processes [11, 13, 16, 19–25], its role in the pathogenesis of KC has not been fully explored from a genetic molecular mechanism perspective.

**Figure 1:**
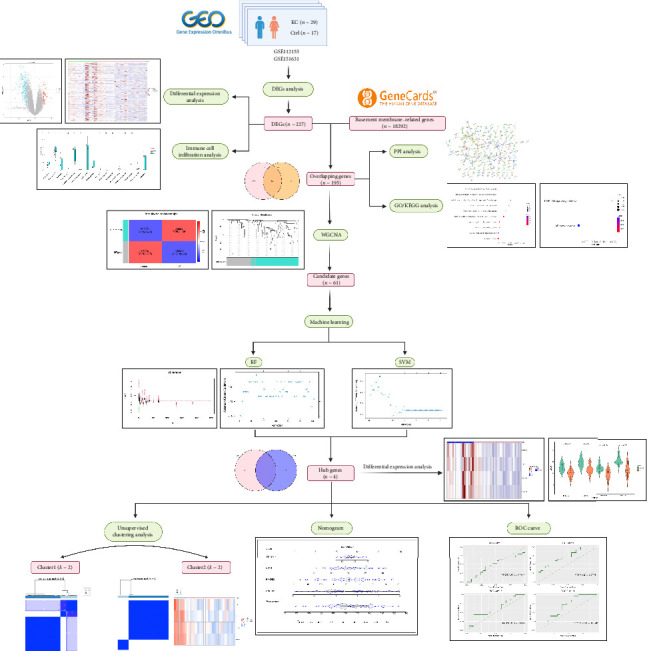
Flowchart of the entire research process.

Consequently, this study aimed to enhance early diagnosis rates and explore novel treatment strategies for KC from this aspect. In this research, we integrated two datasets from the Gene Expression Omnibus (GEO) database to screen out DEGs related to KC. Immunoinfiltration analysis helped assess the role of inflammatory factors in the disease. Then, we performed an intersection analysis between DEGs and BM-related datasets and carried out protein–protein interaction (PPI) and functional enrichment analysis. Weighted gene coexpression network analysis (WGCNA) identified the module most relevant to the development and progression of KC, and machine learning algorithms like random forest (RF) and support vector machine (SVM) helped further select hub genes. Through unsupervised clustering analysis, we validated the significance of these hub genes in distinguishing disease states while discovering two distinct subclasses of KC, which can aid targeted therapy studies. Hub genes' diagnostic potential was confirmed by nomogram and receiver operating characteristic (ROC) curve analysis. Eventually, we determined three key genes with some degree of diagnostic efficacy and considered them as prospective targets.  Figure 1 illustrates the research workflow.

## 2. Methods

### 2.1. Data Collection

We downloaded three datasets related to KC from the GEO database (https://www.ncbi.nlm.nih.gov/geo/): GSE112155, GSE151631, and GSE77938, which contained KC patients and healthy groups. Among them, GSE77938 serves as an external validation dataset. Detailed information of the above datasets can be found in Table 1.

### 2.2. Data Processing and DEGs Screening

In this study, we merged the GSE112155 and GSE151631 databases and applied the “edgeR” package (version 4.0.2) to filter out low-expressed genes [26]. We then utilized the “sva” package (version 3.50.0) [26, 27] to remove batch effects. To screen DEGs between KC and normal samples in the datasets, we performed differential gene analysis on the log2-transformed data using the R package “limma” (version 3.58.1) [28]. DEGs were selected based on the criteria of |log_2_FC| > 1 and *p* < 0.05 [29]. Additionally, we draw a volcano plot of DEGs to visualize the differential expression of DEGs.

We performed preprocessing on BM-related genes obtained from the GeneCards database (https://www.genecards.org/). Specifically, BM-related genes were filtered to retain only protein-coding genes, then identifying the overlapping genes between DEGs in KC and BM-related genes using a “VennDiagram” package (Version 1.7.3).

### 2.3. WGCNA

WGCNA is a powerful bioinformatics analysis method utilized for the identification of crucial modules associated with diseases, the exploration of internal connections between modules and their association with external phenotypes, and the screening of important pathogenesis or potential therapeutic targets [30].

Specifically, in our study, we first preprocessed the sample data and identified outlier samples by hierarchical clustering (hclust) of the gene expression matrix. A sample clustering dendrogram was constructed using average linkage hierarchical clustering with Euclidean distance. To remove potential outliers, we established a cut height of 50. This threshold indicated that samples identified as isolated branches or distinct clusters exceeding this value were considered outliers and subsequently excluded from analysis. Additionally, to ensure stability in downstream analysis, we retained only clusters containing at least 10 samples. Subsequently, we selected the optimal soft threshold power for network construction based on the maximum value of the squared correlation coefficient (*R*^2^) so that the constructed network would be more consistent with the characteristics of a scale-free network. We then converted the adjacency matrix into a topological overlap matrix (TOM). According to soft power and mean connectivity, genes with similar expression patterns were categorized into distinct gene modules. The minimum module size and mergeCutHeight were set at 30 and 0.25, respectively. Each module was assigned a unique color. The correlation between each module and KC was examined utilizing a correlation heatmap. Moreover, the strongest relevant gene module was identified as the key module for subsequent analysis. In addition to performing correlation analysis, including module membership (MM) which means the association of each gene and specific modules, gene significance (GS) which refers to the correlation between modules and phenotypes, and the relationship between MM and GS were summarized. In the key modules, we filtered genes by selecting those with MM greater than 0.8 and GS greater than 0.2 as candidate genes.

### 2.4. Functional Enrichment Analysis

Gene Ontology (GO) and Kyoto Encyclopedia of Genes and Genomes (KEGG) enrichment analysis annotate genes with functional and pathway information and compare DEGs with the entire genome to identify significantly enriched functions and processes associated with disease.

A DAVID 2021 online analysis tool (https://david.ncifcrf.gov/) was implemented to conduct GO and KEGG functional enrichment analysis to assess gene-related biological processes (BPs), cellular components (CCs), molecular functions (MFs), and gene-related signaling pathways. The cutoff criterion for significant enrichment was set as *p* < 0.05.

### 2.5. Construction of PPI Networks

The STRING database (https://string-db.org/) was exploited to establish PPI networks, which Cytoscape software (version 3.8.2) then visualized. The cytoHubba was conducted to score each node gene using four typical algorithms: maximal clique centrality (MCC), maximum neighborhood component (MNC), degree correlation (Degree), and edge percolated component (EPC). Finally, the intersection of the top 30 genes from each algorithm was taken as one of the criteria for validating hub genes.

### 2.6. Machine Learning for Selection of Diagnostic Biomarkers of KC

RF is a machine learning technology that classifies or regresses variables by constructing multiple decision trees. It applies the bootstrap method to obtain multiple different training sets by sampling the original data with replacement. Then, a decision tree is built for each training set, and the final model aggregates the outputs of multiple trees to improve robustness and accuracy. RF is particularly well-suited for high-dimensional biological data, as it can rank feature importance, handle collinearity, and reduce the risk of overfitting [31]. SVM-recursive feature elimination (SVM–RFE) analysis is a supervised machine learning algorithm for selecting the optimal feature genes, particularly in high-dimensional datasets like gene expression data. SVM-RFE systematically removes noninformative features, enhancing classification performance and improving model interpretability. It has been widely applied in genomic studies due to its ability to optimize biomarker selection [32, 33].

In this study, we selected RF and SVM for their effectiveness in high-dimensional gene expression analysis [34]. The dataset was randomly divided into training and test datasets based on the ratio of 7:3. RFE was subsequently employed to choose the optimal subset of diagnostic biomarkers in each model. The parameter “Number of Features to Select' was consistently defined as a sequence ranging from one feature up to the total number of features minus one. This approach enabled systematic evaluation of various feature subsets. During RFE, we leveraged classification accuracy as the performance evaluation metric, assessed via 5-fold repeated cross-validation. The subset of features exhibiting the highest accuracy was selected. Afterward, the importance of these selected genes was further evaluated and visualized, with their relative contributions to model performance quantified and ranked. Features with lower importance scores were excluded from further analysis. The same procedure was consistently applied to both RF and SVM models. Ultimately, to increase the reliability of the selected biomarkers and reduce potential method-specific biases, we considered the intersection of genes identified by both methods as hub genes for subsequent analyses [29, 35]. During these processes, we applied the “randomForest” package (version 4.7-1.1), “e1071” package (version 1.7–13), and “caret” package (version 6.0–94) in R.

### 2.7. Classification of BM-Related KC Clusters

Consensus clustering is a kind of unsupervised clustering method of R package ConsensusClusterPlus Version 1.66.0, which is based on resampling to determine the optimal number of clusters, k [36]. We adopted the K-means clustering algorithm and repeated it 500 times under the condition of pltem = 0.8 to verify the stability of subgroups.

Uniform manifold approximation and projection (UMAP) is a nonlinear dimensionality reduction algorithm which was employed to map high-dimensional probability distributions to a low-dimensional space by “umap” package [37] in the R programming language, achieving the goal of dimensionality reduction.

By following the aforementioned steps, we aim to validate whether the hub genes can be used to determine the disease status [38]. Finally, we use a heatmap to analyze the expression levels of hub genes among different subgroups, which helps to better identify therapeutic targets.

### 2.8. Nomogram Model Construction

The nomogram [39] integrates multiple predictors to express the interrelationship between variables in the prognostic model and validate the importance of genes in diseases through the “rms” R package.

In addition, the “ggplot2,” “ggsignif,” and “ComplexHeatmap” packages were used to visualize the gene expression levels of hub genes between the healthy control group and KC group in the form of violin plot and heatmap.

### 2.9. Immune Cell Infiltration Analysis

CIBERSORT [40] employs a deconvolution algorithm to estimate the composition and abundance of immune cells in the microenvironment. In the present research, we calculated the proportion of 22 immune cell species in KC patients and control samples in DEGs using the CIBERSORT algorithm, which was presented as a boxplot.

### 2.10. Assessment of the Diagnostic Significance of Hub Genes in KC

To assess the discriminative capacity of the hub genes for non-KC controls and patients with KC, we used three datasets: GSE112155, GSE151631, and GSE77938. Using the “GEOquery” package, we obtained the corresponding gene expression matrix for the GSE77938 database. Subsequently, we plotted the ROC curves and calculated the area under the curve (AUC) values using the “ROCR” and “gridExtra” packages. The ROC curve analysis was used to assess the diagnostic value of hub genes by reflecting the relationship between sensitivity (TPR, true positive rate) and specificity (FPR, false positive rate) at various thresholds [41]. The area under the ROC curve is called the AUC. A larger AUC value indicates a higher accuracy of the predictive model. Genes with an AUC value > 0.6 were considered to have potential diagnostic significance and were defined as diagnostic biomarkers.

### 2.11. Statistical Analysis

All data processing and analysis were conducted using R version 4.3.2. Between-group comparisons were conducted via the Wilcoxon rank-sum test. Pearson correlation analysis was employed to calculate correlation coefficients. Based on logistic regression modeling, a nomogram was constructed to further assess the importance of corn genes in disease identification. *p* < 0.05 was regarded as statistically significant.

## 3. Results

### 3.1. Identification of DEGs Associated With BM

**Figure 2:**
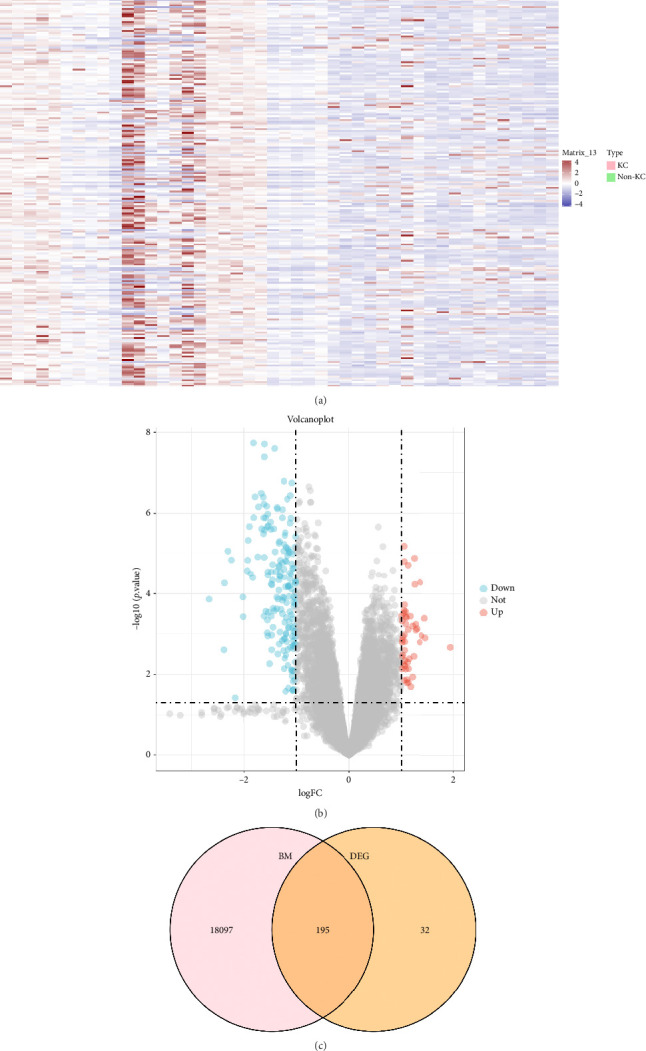
Identification of DEGs related to BM. (a) Heatmap of DEGs between KC and health controls. (b) Volcano plot of DEGs, where upregulated genes are denoted by red dots, downregulated genes are denoted by blue dots, and genes with no significant difference are represented by grey dots. (c) Venn diagram depicting the overlap between BM genes and DEGs. The intersection represents the identification of BM genes from DEGs.

We extracted 46 samples based on the GEO database. A total of 12,614 KC-related genes were identified after preprocessing. The differential analysis identified 227 DEGs between KC and non-KC groups. The heat map clearly shows the expression of these 227 DEGs in KC group and non-KC group (Figure 2(a)). As shown in the volcano plot (Figure 2(b)), 49 of the DEGs were upregulated and 178 were downregulated in cornea tissues. Among these DEGs, 195 genes overlapped with the 18,292 BM-related genes from the GeneCards database (Figure 2(c)).

### 3.2. WGCNA to Screening Crucial Modules and Candidate Genes

**Figure 3:**
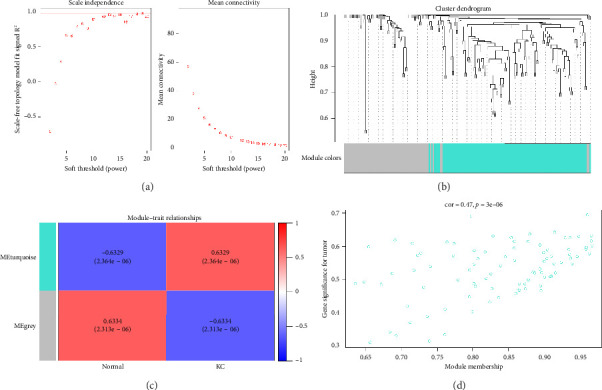
Screening crucial modules and candidate genes based on WGCNA. (a) Examination of the scale-free exponent and the mean connectivity across different soft threshold powers. (b) Dendrogram of all overlapping genes clustered based on average hierarchical clustering and dynamic tree clipping, each module distributed a unique color. (c) Heatmap of the correlation analysis between the module signature genes and KC. (d) Scatter plot of relationship between MM and GS in KC.

When the soft threshold was 19, the scale-free network (*R*^2^ = 0.969) and connectivity exhibited maximum efficiency (Figure 3(a)). The above 195 genes were classified into two modules (Figure 3(b)). The turquoise module, which was chosen as a KC-related module for further analysis, comprised 122 genes and exhibited a strong correlation with KC (*R* = 0.6329) (Figure 3(c)). Furthermore, we also confirmed there was a highly significant correlation between MM and GS in this module (Cor = 0.47, *p*=3*e* − 05) (Figure 3(d)). Finally, 61 genes were selected as candidate genes.

### 3.3. GO and KEGG Analysis of Samples

**Figure 4:**
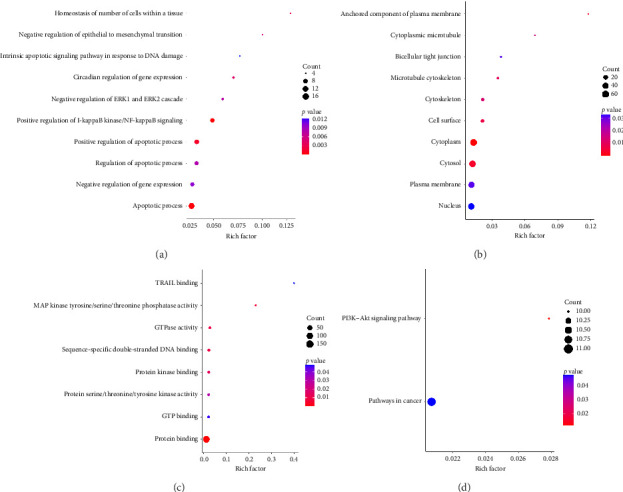
Functional enrichment analysis results. (a–d) The bubble charts of GO annotations (BP, CC, and MF) and KEGG signal pathways for 195 intersecting genes.

For BP, 195 overlapping genes were mostly engaged in positive regulation of I-kappaB kinase/NF-kappaB signaling (*p*=0.0001274293), apoptotic process (*p*=0.0002163301), and positive regulation of apoptotic process (*p*=0.0012026415) (Figure 4(a)). DEGs related to BM in KC have been localized to cytoplasm (*p*=0.0001089293), cytosol (*p*=0.0033262310), anchored component of plasma membrane (*p*=0.0034992529), and other structures in CC (Figure 4(b)). The overlapping gene changes associated with MF included protein binding (*p*=2.019459e − 05), MAP kinase tyrosine/serine/threonine phosphatase activity (*p*=6.870702e − 03), sequence-specific double-stranded DNA binding (*p*=8.981771e − 03), and other functions (Figure 4(c)). As shown in Figure 4(d), KEGG signal pathway enrichment analysis results showed that the 195 genes were particularly abundant in the PI3K–AKT signaling pathway (*p*=0.01178047) and pathways in cancer (*p*=0.04736040).

### 3.4. Gene Correlation Analysis Based on PPI Networks

The constructed PPI network (confidence score = 0.4) contained 195 nodes and 153 edges (Supporting Figure 1). Using MCC, MNC, Degree, and EPC algorithms, we identified 20 common genes, namely, CD8A, CCR7, MCL1, UBC, BCL2L11, PRDM1, CCR2, RXRA, UBE2V1, HLA-B, KLF4TNFRSF10B, ID2, BCL10, RIPK2, BCL2A1, TUBA1A, BHLHE40, NR4A2, and MAP2K3. Further details of the top 30 genes from each algorithm are provided in Supporting Table 1.

### 3.5. Optimization of Hub Genes Filtering

**Figure 5:**
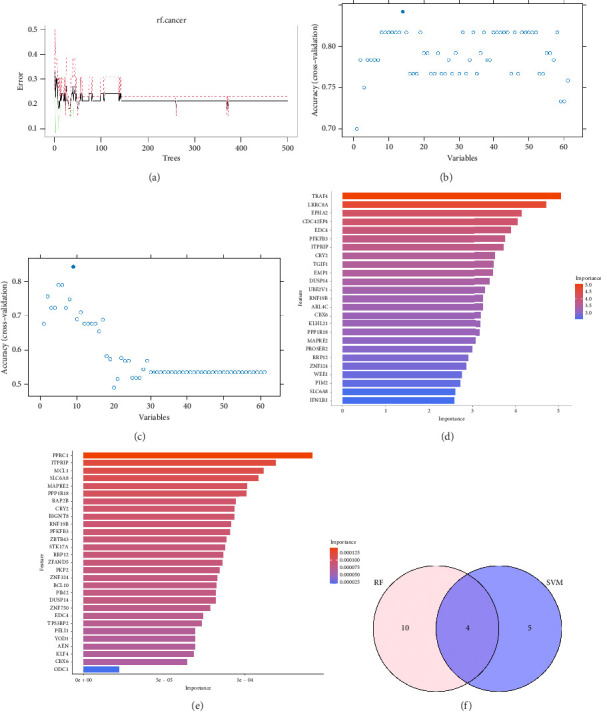
Screening for potential KC biomarker by machine learning. (a, b) Feature genes were selected by the RF algorithm. (c) Characteristic genes chosen using the SVM algorithm. (d, e) The bar charts illustrating the importance of genes extracted by the RF and SVM machine learning methods, respectively. (f) Venn diagram displaying the hub genes intersected by SVM and RF algorithm.

Based on the 61 candidate genes, RF, RFE, and SVM algorithms identified an optimal biomarker subset. The parameter diagram of the algorithm screening is shown in Figure 5. As shown in Figure 5(a), when the number of factors was 9, the classifier had the minimum error (0.2188). In RF–RFE and SVM–RFE algorithms, we screened 14 and 9 DEGs (Figures 5(b) and 5(c)), respectively. Moreover, genes selected by each method were ranked according to their feature importance scores (Figures 5(d) and 5(e)). Intersection analysis identified four overlapping DEGs (6-phosphofructo-2-kinase/fructose-2,6-biphosphatase 3 [PFKFB3], cryptochrome 2 [CRY2], ring finger protein 19B [RNF19B], and protein phosphatase 1 regulatory subunit 18 [PPP1R18]) as the final optimal biomarker set (Figure 5(f)).

### 3.6. Consensus Clustering for Identifying Sample Subclasses

**Figure 6:**
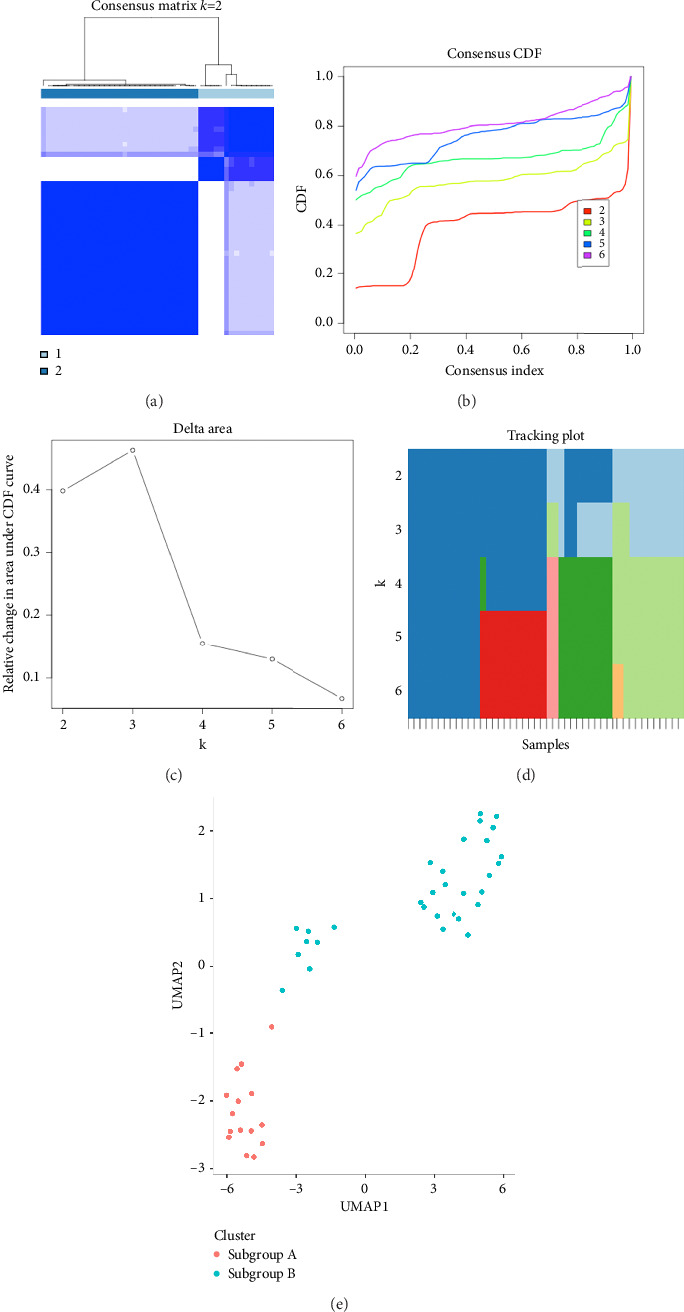
Consensus clustering analysis utilizing hub genes. (a) Consensus clustering matrix when *k* = 2. (b) CDF curve for *k* = 2–6 is shown. (c) Assist in determining the optimal grouping based on the inflection point in the delta area plot. (d) The tracking plot showing the subtyping of samples under different *K* values. (e) UMAP indicating the diagnostic efficacy of four potential signatures in KC.

Based on the four BM-related KC hub genes, consensus clustering identified distinct subgroups among the 46 samples. They were categorized into two to six subclasses. After comprehensive consideration, *k* = 2 was determined as the optimal number of clusters (Figures 6(a), 6(b), 6(c), 6(d)). When *k* = 2, the consensus matrix heatmap exhibited relatively clear and distinct boundaries (Figure 6(a)) and the cumulative distribution function (CDF) plot displayed the minimum fluctuation (Figure 6(b)). Our UMAP results also indicated that patients could be effectively distinguished (Figure 6(e)).

**Figure 7:**
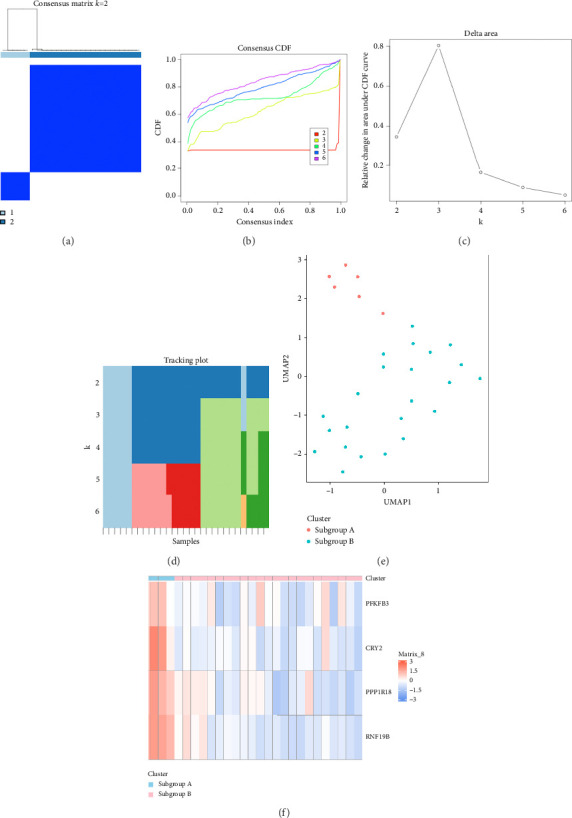
Identification of the KC subtypes. (a–d) When *k* = 2, it exhibits the strongest intracluster relationship. (e) UMAP supporting stratification into two KC subclasses. (f) Hub gene expression heatmap for the two KC subgroups. High expression is depicted in red, low expression in blue, and no difference in white.

Furthermore, KC samples were optimally divided into two clusters (*k* = 2), reflecting the strongest intracluster similarity (Figures 7(a), 7(b), 7(c), 7(d)). Both UMAP and a heatmap revealed significant variances in gene expression profiles between the two subgroups (Figures 7(e) and 7(f)). From Figure 7(f), it can be observed that these four hub genes are majority distributed in subgroup A, which is beneficial for the subsequent targeted identification and treatment of genes.

### 3.7. Validation of the Feature Gene Diagnostic Signature for KC

**Figure 8:**
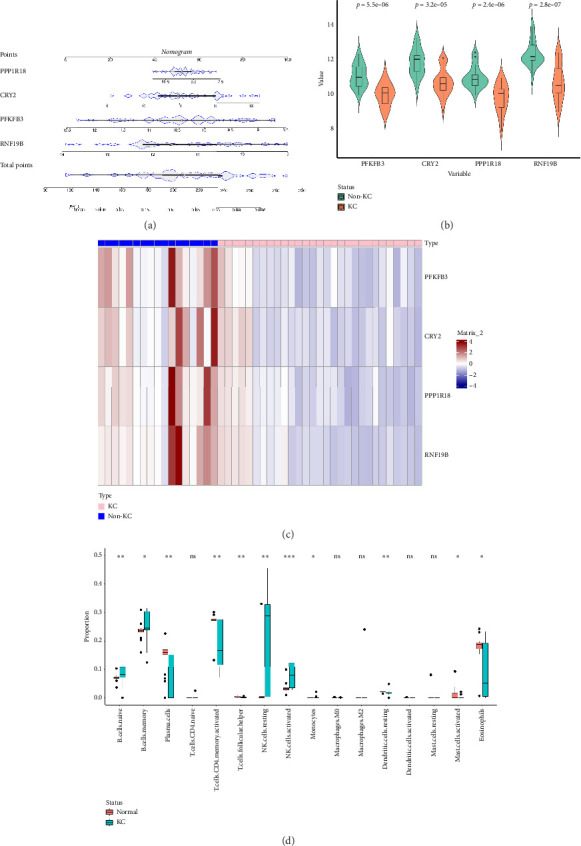
Construction of a nomogram and differential expression analysis of key genes as well as immune analysis. (a) A nomogram model to predict the KC progression was established based on four characteristic genes. (b) Violin plot of potential diagnostic markers between KC and health controls. (c) Heatmap of key genes between KC and non-KC groups. (d) Boxplot of different immune cells between KC and health controls (ns indicates no significance; ^∗^*p* < 0.05, ^∗∗^*p* < 0.01, and ^∗∗∗^*p* < 0.005).

In accordance with the illustration provided in Figure 8(a), the four hub genes all had a potential effect to predict the risk of KC. Similarly, these hub genes showed different expressions between the KC and non-KC tissues, with significantly higher expression levels in the normal groups compared to the disease groups (Figures 8(b) and 8(c)).

### 3.8. Association Between KC and Immune Microenvironment

The CIBERSORT analysis showed that 11 types of immune cells were demonstrated to be comparable between KC and control samples. Among them, 7 cells showed remarkable expression differences between the two groups, including naive B cell, plasma cells, activated memory CD4 T cell, T follicular helper cells, resting natural killer (NK) cell, activated NK cell, and resting dendritic cells. Specifically, we found that naive B cell, resting NK cell, and activated NK cell were dramatically higher in the KC group (Figure 8(d)).

### 3.9. ROC Curve Analysis

**Figure 9:**
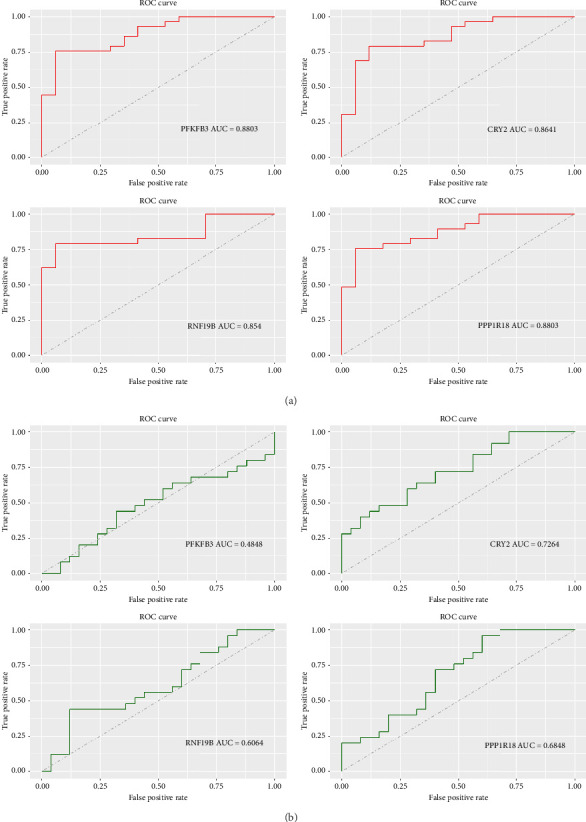
ROC curve validating the diagnostic model. (a) ROC curves were plotted to assess the predictive ability of hub genes in internal datasets. (b) ROC curves demonstrating the diagnostic performance of the feature genes in the GSE77938 datasets. The AUC value refers to the area under the curve.

As shown in Figure 9(a), the AUC values were 0.8803 for PFKFB3, 0.8641 for CRY2, 0.854 for RNF19B, and 0.8803 for PPP1R18 in internal datasets. In the external validation dataset (GSE77938), the AUC values were 0.4848 for PFKFB3, 0.7264 for CRY2, 0.6064 for RNF19B, and 0.6848 for PPP1R18 (Figure 9(b)). To some degree, these results also indicated that the three genes (CRY2, RNF19B, and PPP1R18) have good predictive performance in the pathogenesis of KC.

## 4. Discussion

KC is a primary corneal degenerative disease that can lead to highly irregular myopia and astigmatism. The incidence of this disease is higher in children and teenagers, and it tends to manifest more severe changes, resulting in more serious visual impairment and an increased likelihood of requiring corneal transplantation. Without prompt and suitable intervention, there is a risk of blindness. While it was once thought to be a noninflammatory condition, recent research suggests a potential link to inflammatory mediators [42]. In addition, factors such as genetics and oxidative stress are believed to play a role in the development of the disease [43]. Nevertheless, the exact pathogenesis of KC remains under ongoing investigation. Furthermore, there are restrictions in early diagnosis and treatment approaches. Hence, there is a need for in-depth research and refinement of biomarkers to advance both diagnostic accuracy and therapeutic strategies for KC management.

Nowadays, accumulating evidence suggested that BM plays an important role in kinds of diseases. For instance, misalignment or thickness changes in the glomerular BM impact its filtration barrier, which results in impaired kidney function [19]. Hyperglycemia can induce alterations in BM protein turnover, and thus, diabetes-related complications are frequently associated with BM changes [13]. The thickening of vascular BM compromises the blood–retinal barrier and the blood–brain barrier, leading to conditions such as diabetic retinopathy, Alzheimer's disease, and stroke [20, 21]. It has been discovered that BM is also involved in tumor genesis and development through mechanisms including overexpression of its components (especially laminin), attenuation of T-cell activation, and promotion of cancer cell diffusion due to its structural and functional incompleteness [11, 13, 22–24]. Plus, abnormalities in corneal epithelial BM are linked to various ocular diseases like recurrent epithelial erosions, lattice corneal dystrophy, and bullous keratopathy [16].

Most importantly, it has been suggested that BM may be associated with the onset of KC. On the one hand, this involves alterations in its composition, particularly the aberrant expression of fibronectin, Type IV collagen, and laminin. The absence of fibronectin in BM and its excessive expression in the stroma are two potential factors contributing to KC. The former may compromise the integrity of BM, while the latter may promote a fibrotic reaction within the stroma leading to corneal scarring [14]. Type IV collagen plays a crucial role in corneal development and wound healing, as well as being a major determinant of BM hardness. Through its uneven hardness, it changes the resistance of BM to the growth of its covering epithelium, which facilitates tissue shaping [13]. However, increased activity of collagenase in KC patients disrupts the balance between collagen synthesis and degradation, which leads to the weakening and thinning of corneal stroma [44]. Defects in the laminin gene are also associated with this disease manifestation. On the other hand, the rupture of BM leads to release proinflammatory molecules which potentially contribute to KC progression through inflammatory pathways. Furthermore, the dysfunction of the BM barrier in controlling stromal cell-derived growth factor may be a vital factor in the corneal epithelium proliferation and thickening observed in this condition [16]. Despite evidence implicating abnormal BM as a pathological mechanism underlying KC, the precise genes and biological functions are yet to be identified.

In our research, we employed advanced bioinformatics techniques to identify four crucial genes linked to the BM: PFKFB3, CRY2, RNF19B, and PPP1R18. Through consensus clustering analysis, we not only reaffirmed the importance of these genes in diagnosing KC but also delineated two subtypes of KC (subtype A and subtype B). Notably, we observed a predominant distribution of these four key genes in subtype A. Besides, we evaluated the diagnostic efficacy of these genes with nomogram. Additionally, we noted a significant increase in the expression levels of these genes in the non-KC group. Finally, ROC curve analysis revealed that CRY2, RNF19B, and PPP1R18 have AUC values exceeding 0.6 in both internal and external datasets, indicating their diagnostic utility.

CRY2 encodes a flavin adenine dinucleotide-binding protein [45]. As a circadian gene, CRY2's abnormal regulation leads to the disturbance of circadian rhythm, which can induce the development of cancers (like breast cancer) by affecting specific physiological functions such as signal transduction, cell division, and growth [46, 47]. Additionally, CRY2 plays a crucial role in sleep disorders, including its association with familial advanced sleep phase (FASP) [48]. A missense mutation in the CRY2 gene alters its stability, resulting in a shortened circadian rhythm and reduced phase shifts to early-night light pulses, which in turn affects sleep–wake behavior in humans. Studies have also shown that dysregulation of CRY2 expression can increase susceptibility to depression [49, 50]. At the same time, metabolic processes are also influenced by circadian oscillations. Recent research has revealed CRY2's involvement in regulating fasting blood glucose levels and its significant relevance to Type 2 diabetes [51–53]. But little is known about the role of CRY2 in ocular diseases. We propose for the first time that CRY2 may be a potential biomarker for KC. Given the heatmap showing a significant increase in its expression in the non-KC group, we hypothesize that the abnormality in CRY2 may impact circadian rhythmicity, leading to metabolic disorders, influencing the normal expression of proteins in corneal tissue, and thus undermining its morphological and structural integrity. Although our results demonstrate that CRY2 has a high diagnostic value in the analysis of nomogram and ROC curve, it has not been verified by PCR and other experiments. Accordingly, further research is necessary to confirm its potential role as a reliable biomarker for KC.

RNF19B is an E3 ubiquitin–protein ligase. Studies have indicated that RNF19B can modify the stability and function of target proteins through ubiquitination, influencing the activity of NK cells as well as the activation and regulation of inflammatory signaling pathways. This changes the immune system's ability to clear pathogens and tumor cells, and the body's response to inflammatory diseases such as infection and tissue damage [54]. Niu and Sun et al. proved an obvious correlation between immune-inflammatory factors and KC [29, 55]. Thus, we speculate that RNF19B promotes the progress of KC by regulating immune responses and inflammatory reactions.

PPP1R18 can target enzymes to different cellular locations and alter their activity toward specific substrates. For example, targeting PP1 to the F-actin cytoskeleton, which varies the formation of actin ring and the performance of bone resorption activity [56, 57]. In our study, PPP1R18 showed promising capabilities in separating patients with KC from normal individuals. Overall, these genes may serve as important markers for KC, but it is essential for further research to clear their underlying molecular mechanisms.

PFKFB3 is a bifunctional enzyme that is indispensable for cell cycle progression (such as glycolysis, cell proliferation, and adhesion) and the prevention of apoptosis, which makes it as a promising novel target for the therapeutic arsenal against cancer [58, 59]. Whereas, in this work, it regrettably did not garner sufficient validation within external datasets, we currently withhold its designation as a predictive factor for KC occurrence, pending further extensive research to delineate its role within the context of KC pathology.

With CIBERSORT, we conducted a comprehensive assessment of the meaning of immune-infiltrating cells in KC and identified 11 cells that expressed significantly different in the non-KC and KC groups, once again making sure the critical role of immune factors in KC development. Of particular note is the proportions of naive B cells, resting NK cells, and activated NK cells were apparently increased in KC patients. Recently, D'Souza et al. [42] conducted single-cell RNA sequencing on corneal tissue from KC patients and healthy controls, revealing a significant increase in the proportion of activated neutrophils, NK cells, and γδT cells in KC patients. Interestingly, within the KC group, there was an imbalance between neutrophils and NK cells, with a higher proportion of NK cells. Given that neutrophils and NK cells have opposing effects on corneal thickness regulation, it is suggested that there may be a negative correlation between NK cells and corneal thickness. Further exploration, both in vivo and in vitro, is warranted for the other immune cell types, as their involvement in KC has not been previously documented.

According to the enrichment analysis, these 195 intersecting genes are mainly involved in the BP of I-kappaB kinase/NF-kappaB signaling and apoptosis, while also implicating the PI3K–AKT signaling pathway. Multiple studies have indicated that the I-κB kinase/NF-κB signaling pathway plays a crucial role in immune response, inflammation, and apoptosis [60], but its relationship with KC has not been clarified. Kaldawy et al.'s research has pointed out that apoptosis plays a critical role in corneal thinning in KC patients [61]. Additionally, differential expression of apoptotic genes has been identified in KC [62]. Furthermore, analysis of tears or corneal tissues from KC patients has revealed the involvement of apoptosis and scar formation in the disease progression [7]. Activation of the PI3K–AKT signaling pathway promotes the antiapoptotic, anti-inflammatory, proliferative, migratory functions of corneal epithelial cells, and wound healing [63]. Former studies have suggested that the PI3K–AKT pathway is one of the pathways involved in the pathogenesis of KC, but its activity is downregulated in tissues with KC [64]. These perspectives are consistent with the conclusions of this study.

It is inevitable that this study has some limitations. Firstly, our study is based on the secondary mining and analysis of public datasets. Although we used another dataset for expression validation, further experimental validation is needed before it can be applied to clinical practice. Secondly, the limited sample size of the public dataset may lead to potential biases in predicting the progression of KC. Additionally, issues with the dataset may have resulted in an insufficient evaluation of the predictive performance of these four diagnostic markers, which could be related to their AUC values not reaching 0.8. Therefore, it is necessary to incorporate more accurate and extensive clinical data for further validation in order to improve the accuracy of the model.

In summary, through a series of bioinformatics methods, we successfully identified three key genes (CRY2, RNF19B, and PPP1R18) related to BM. This represents a significant breakthrough in advancing our understanding of the pathogenesis of KC and provides new perspectives and insights for the diagnosis and treatment of this disease.

## Figures and Tables

**Table 1:** Information of the three datasets utilized and their features.

Dataset	Database	Platform	Species	Tissue	KC	Non-KC
GSE112155	GEO	GPL18573	*Homo sapiens*	Corneal epithelial cells	10	10
GSE151631	GEO	GPL16791	*Homo sapiens*	Corneas	19	7
GSE77938	GEO	GPL18460	*Homo sapiens*	Corneas	25	25

*Note:* “KC” represents keratoconus, “Non-KC” represents healthy control, and “GEO” represents Gene Expression Omnibus.

## Data Availability

The data for the article are sourced from the GEO database (https://www.ncbi.nlm.nih.gov/geo/): GSE112155, GSE151631, and GSE77938.

## Supporting Information

Supporting Information 1 Supporting Figure 1: The PPI network based on the overlapping genes showing potential interactions among relevant molecules. The network nodes represent proteins, and the edges represent the protein–protein associations.

Supporting Information 2 Supporting Table 1: Top 30 genes identified by four algorithms in protein–protein interaction network analysis.
