# Supplementary material for: Screening and Identification of Basement Membrane–Related Gene Signatures for Diagnosis in Keratoconus Through WGCNA and Machine Learning
Source: J Ophthalmol. 2025 Jun 1;2025:7107888. doi: 10.1155/joph/7107888 (PMC12145936; doi:10.1155/joph/7107888)
Supplement: Supporting Information 1 — Supporting Figure 1: The PPI network based on the overlapping genes showing potential interactions among relevant molecules. The network nodes represent proteins, and the edges represent the protein–protein associations. [file 7107888.f1.docx]

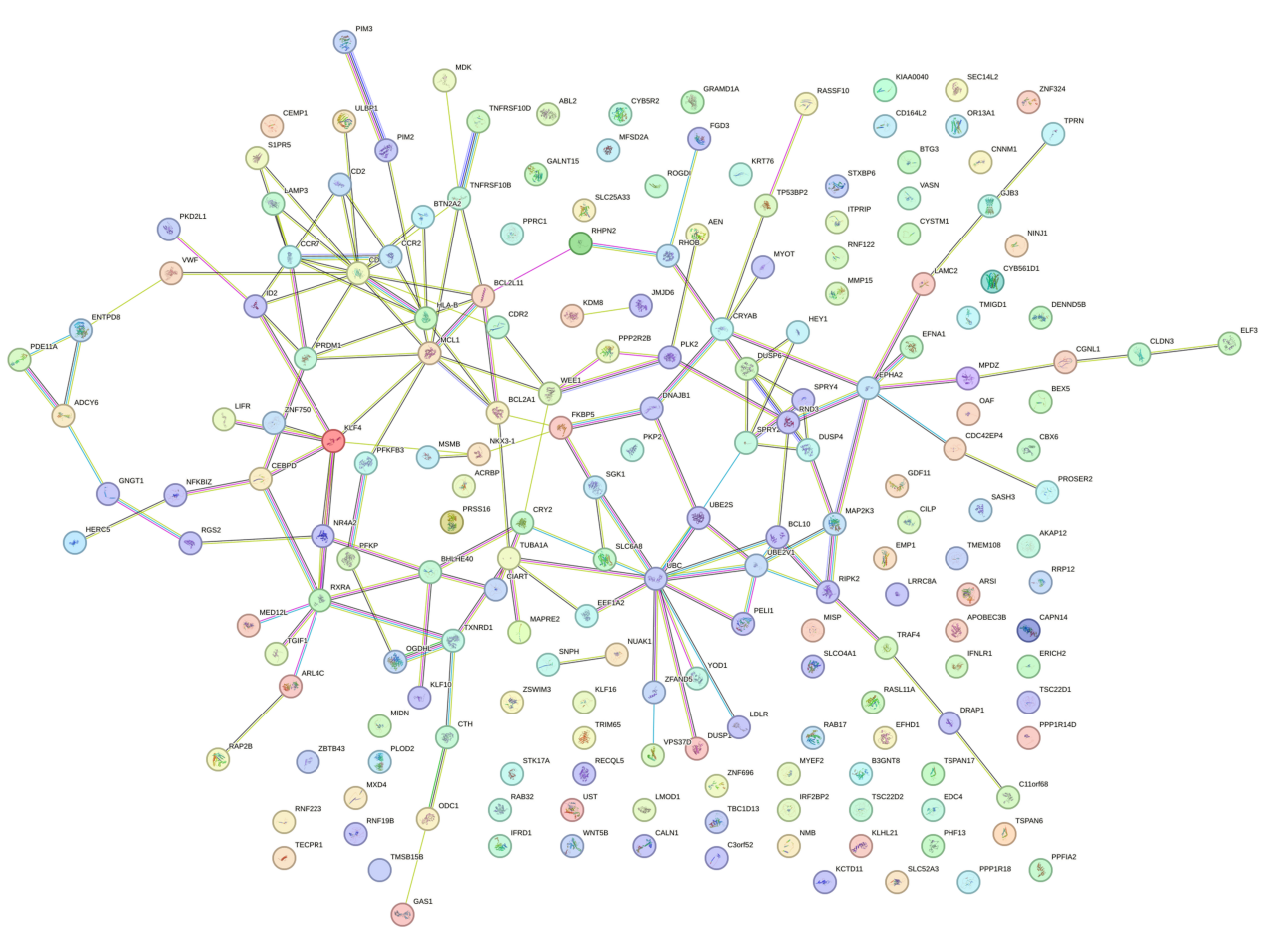


**Supplementary Fig 1.** The PPI network based on the overlapping genes showing potential interactions among relevant molecules. The network nodes represent proteins, and the edges represent the protein–protein associations.
