# Supplementary material for: Screening and Identification of Basement Membrane–Related Gene Signatures for Diagnosis in Keratoconus Through WGCNA and Machine Learning
Source: J Ophthalmol. 2025 Jun 1;2025:7107888. doi: 10.1155/joph/7107888 (PMC12145936; doi:10.1155/joph/7107888)
Supplement: Supporting Information 2 — Supporting Table 1: Top 30 genes identified by four algorithms in protein–protein interaction network analysis. [file 7107888.f2.docx]

**Supplementary Table 1. Top 30 genes identified by four algorithms in protein-protein interaction network analysis.**

| Rank | MCC | | MNC | | Degree | | EPC | |
| --- | --- | --- | --- | --- | --- | --- | --- | --- |
|  | Name | Score | Name | Score | Name | Score | Name | Score |
| 1 | CD8A | 39 | CD8A | 12 | CD8A | 15 | CD8A | 41.952 |
| 2 | CCR7 | 22 | CCR7 | 8 | UBC | 14 | MCL1 | 41.579 |
| 3 | MCL1 | 18 | UBE2V1 | 6 | MCL1 | 9 | CCR7 | 41.092 |
| 4 | UBC | 16 | MCL1 | 5 | RXRA | 8 | PRDM1 | 40.825 |
| 5 | BCL2L11 | 15 | BCL2L11 | 5 | CCR7 | 8 | BCL2L11 | 40.099 |
| 6 | PRDM1 | 13 | PRDM1 | 5 | KLF4 | 8 | BCL2A1 | 39.292 |
| 7 | CCR2 | 12 | RXRA | 4 | EPHA2 | 7 | KLF4 | 39.232 |
| 8 | RXRA | 10 | UBC | 4 | UBE2V1 | 6 | ID2 | 38.842 |
| 8 | UBE2V1 | 10 | HLA-B | 4 | BCL2L11 | 6 | HLA-B | 38.757 |
| 10 | HLA-B | 9 | SPRY2 | 4 | PRDM1 | 6 | UBC | 38.496 |
| 10 | SPRY2 | 9 | DUSP6 | 4 | BCL2A1 | 5 | CCR2 | 37.827 |
| 10 | DUSP6 | 9 | CCR2 | 4 | HLA-B | 5 | RXRA | 36.823 |
| 10 | KLF4 | 9 | BCL10 | 3 | TUBA1A | 5 | TNFRSF10B | 36.366 |
| 14 | TNFRSF10B | 8 | RIPK2 | 3 | TNFRSF10B | 5 | CD2 | 35.936 |
| 14 | ID2 | 8 | DUSP4 | 3 | SPRY2 | 5 | TUBA1A | 35.347 |
| 16 | DUSP4 | 7 | TNFRSF10B | 3 | DUSP6 | 5 | UBE2V1 | 34.865 |
| 16 | EPHA2 | 7 | SPRY4 | 3 | BHLHE40 | 5 | CEBPD | 34.537 |
| 18 | SPRY4 | 6 | NR4A2 | 3 | ID2 | 5 | WEE1 | 33.547 |
| 18 | CD2 | 6 | CD2 | 3 | WEE1 | 5 | BHLHE40 | 33.452 |
| 20 | BCL10 | 5 | ID2 | 3 | BCL10 | 4 | BCL10 | 33.427 |
| 20 | RIPK2 | 5 | KLF4 | 3 | CRYAB | 4 | NR4A2 | 33.402 |
| 20 | BCL2A1 | 5 | PELI1 | 2 | RIPK2 | 4 | CRY2 | 33.026 |
| 20 | TUBA1A | 5 | BCL2A1 | 2 | TXNRD1 | 4 | FKBP5 | 31.745 |
| 20 | BHLHE40 | 5 | TUBA1A | 2 | FKBP5 | 4 | LAMP3 | 30.744 |
| 20 | NR4A2 | 5 | UBE2S | 2 | DUSP4 | 4 | UBE2S | 30.701 |
| 20 | WEE1 | 5 | PPP2R2B | 2 | MAP2K3 | 4 | BTN2A2 | 30.47 |
| 27 | CRYAB | 4 | MAP2K3 | 2 | NR4A2 | 4 | S1PR5 | 30.267 |
| 27 | TXNRD1 | 4 | BHLHE40 | 2 | CRY2 | 4 | MAP2K3 | 29.861 |
| 27 | FKBP5 | 4 | HEY1 | 2 | CCR2 | 4 | TXNRD1 | 29.494 |
| 27 | MAP2K3 | 4 | CIART | 2 | CEBPD | 4 | RIPK2 | 28.859 |

Note: “MCC” represents maximal clique centrality, “MNC” represents maximum neighborhood component, “Degree” represents degree correlation, “EPC” represents edge percolated component.
